# Development of a Transformable Fast-Flowering Mini-Maize as a Tool for Maize Gene Editing

**DOI:** 10.3389/fgeed.2020.622227

**Published:** 2021-01-11

**Authors:** Morgan E. McCaw, Keunsub Lee, Minjeong Kang, Jacob D. Zobrist, Mercy K. Azanu, James A. Birchler, Kan Wang

**Affiliations:** ^1^Department of Agronomy, Iowa State University, Ames, IA, United States; ^2^Crop Bioengineering Center, Iowa State University, Ames, IA, United States; ^3^Interdepartmental Plant Biology Major, Iowa State University, Ames, IA, United States; ^4^Interdepartmental Genetics and Genomics Major, Iowa State University, Ames, IA, United States; ^5^Division of Biological Sciences, University of Missouri, Columbia, MO, United States

**Keywords:** *Agrobacterium*-mediated transformation, CRISPR, embryogenic callus, gene editing, transgenesis, *Zea mays*

## Abstract

Maize (*Zea mays* ssp. *mays*) is a popular genetic model due to its ease of crossing, well-established toolkits, and its status as a major global food crop. Recent technology developments for precise manipulation of the genome are further impacting both basic biological research and biotechnological application in agriculture. Crop gene editing often requires a process of genetic transformation in which the editing reagents are introduced into plant cells. In maize, this procedure is well-established for a limited number of public lines that are amenable for genetic transformation. Fast-Flowering Mini-Maize (FFMM) lines A and B were recently developed as an open-source tool for maize research by reducing the space requirements and the generation time. Neither line of FFMM were competent for genetic transformation using traditional protocols, a necessity to its status as a complete toolkit for public maize genetic research. Here we report the development of new lines of FFMM that have been bred for amenability to genetic transformation. By hybridizing a transformable maize genotype high Type-II callus parent A (Hi-II A) with line A of FFMM, we introgressed the ability to form embryogenic callus from Hi-II A into the FFMM-A genetic background. Through multiple generations of iterative self-hybridization or doubled-haploid method, we established maize lines that have a strong ability to produce embryogenic callus from immature embryos and maintain resemblance to FFMM-A in flowering time and stature. Using an *Agrobacterium*-mediated standard transformation method, we successfully introduced the CRISPR-Cas9 reagents into immature embryos and generated transgenic and mutant lines displaying the expected mutant phenotypes and genotypes. The transformation frequencies of the tested genotypes, defined as the numbers of transgenic event producing T1 seeds per 100 infected embryos, ranged from 0 to 17.1%. Approximately 80% of transgenic plants analyzed in this study showed various mutation patterns at the target site. The transformable FFMM line, FFMM-AT, can serve as a useful genetic and genomic resource for the maize community.

## Introduction

Recent years have ushered in rapid advances in precise gene editing technologies such as clustered regularly interspaced short palindromic repeats (CRISPR)-Cas systems (Jinek et al., 2012; Zetsche et al., 2015). The advent of gene editing has placed increased importance on the ability to genetically transform plants. Methods of plant transformation and their difficulty differ greatly between species; what works well for one species may not work at all in other species. In maize, the most successful methods have traditionally relied on transformation of embryogenic callus derived from the scutellum of immature zygotic embryos (IZEs). Although this method has been widely used in maize transformation, few inbred maize lines are capable of readily producing embryogenic callus that can be transformed and regenerated into plants.

Maize embryogenic callus has been traditionally classified as either Type-I: hard, compact, and relatively slow growing; or Type-II: highly friable, relatively fast growing, and with abundant somatic embryos (Tomes and Smith, 1985). Type-I callus response is typically induced by a Murashige and Skoog (MS) based medium and is more common than Type-II. B104 is a popular line for Type-I transformation (Frame et al., 2006; Raji et al., 2018), due to its high percentage (~60%) of genetic similarity to B73 (Liu et al., 2003), which was used to produce the first maize reference genome (Schnable et al., 2009). Success has also been reported in A188 and H99 (Ishida et al., 2003), B114 and Ky21 (Frame et al., 2006) as well as a number of tropical lines (Carvalho et al., 1997; Bohorova et al., 1999; Valdez-Ortiz et al., 2007; Anami et al., 2010; Ombori et al., 2013). Type-II callus in maize is typically induced by an N6-based medium and was originally derived from embryos of A188 or B73 × A188 hybrids (Armstrong and Green, 1985; Tomes and Smith, 1985). A maize genotype with a high Type-II callus induction rate (Hi-II) is one of the most popular and user-friendly lines for transformation (Armstrong et al., 1991). The Hi-II system is a hybrid formed by a cross of lines “Parent A” and “Parent B”. These lines were selected from two independent F2 embryos of an A188 × B73 hybrid with the ability to generate Type-II callus. The regenerant seed (R1) plants were then grown and tested for ~100% Type-II callus formation in half ears and the remaining R2 seed from two plants of each embryo lineage were used to produce sib populations that comprise “Parent A” and “Parent B” (Armstrong et al., 1991).

Fast-Flowering Mini-Maize (FFMM) was developed to accelerate maize genetic research by reducing the long generation time and substantial space requirements of maize (McCaw et al., 2016). Two independent inbred lines, FFMM-A and FFMM-B, were generated using single-seed descent from a modified double-cross hybrid of four early flowering lines. Both lines can go from seed-to-seed in 60 days, producing 5–6 generations per year as compared to the 2–3 generations in traditional lines. Both FFMM lines also require less growth substrate per plant and about four plants can be grown in the same footprint of a traditional maize plant. FFMM also performs well in inexpensive, modular growth chamber setups, which makes it more accessible to researchers without access to greenhouses (Tran and Braun, 2017). FFMM plants are short enough to grow on stackable shelves with a proper lighting system. The original FFMM lines are not capable of genetic transformation by traditional protocols, though they work very well with the QuickCorn Babyboom/Wuschel morphogenic genes technology (Lowe et al., 2016, 2018; Jones et al., 2019; Masters et al., 2020). This morphogenic gene technology, while effective, does have some limitations such as large construct size and restrictive licensing options. The ability to transform FFMM through traditional methods completes this germplasm as an open-source tool for maize genetics research. In this work, we report the breeding and tissue culture efforts toward generation of FFMM lines with a robust ability to produce embryogenic callus from IZEs. We then demonstrate that these lines can be transformed using an *Agrobacterium*-mediated standard transformation method for efficient targeted mutagenesis by a CRISPR-Cas9 system.

## Materials and Methods

### Germplasm Availability, Development, and Greenhouse Care

Fast-Flowering Mini-Maize A (FFMM-A, McCaw and Birchler, 2017) and maize haploid inducer line RWS-GFP (Yu and Birchler, 2016) can be obtained from James A. Birchler at the University of Missouri. Maize high Type-II Parent A (Hi-II A, Armstrong et al., 1991) can be obtained from the Maize Genetics Cooperation Stock Center (http://maizecoop.cropsci.uiuc.edu/).

FFMM-AT lines were generated through introgression of competency to form embryogenic callus in tissue culture from Hi-II A into the FFMM-A genetic background. All plants were grown in a greenhouse set to 28°C, 16 h day/25°C, 8 h night in a soilless substrate (Promix BR or Sun Gro LC1) as described previously (McCaw and Birchler, 2017). Seeds were started in seedling flats to germinate for 9–10 days with only deionized (DI) water supplemented. Once established, the plantlets were moved to 1 gallon pots supplemented with 0.66 g 10% iron chelate (Grow More Inc., CA, USA) and watered to ~50% soil saturation with a 15-5-15 (N-P-K) fertilizer at 200 ppm nitrogen whenever the soil was dry ~2 cm below the surface. Once a tassel was visible in the whorl (~26 days) they were switched back to DI water and kept between ~50–75% soil saturation.

To achieve a well-pollinated ear, ear shoots were bagged when flag leaves emerge and tended every day to trim flag leaves and watch for silks. Shoot bags were marked on the 1st day of silking and silks were trimmed on the second day and re-covered with the top of the bag folded to indicate the cut. Pollination was performed on the following morning as previously described (McCaw, 2017).

After pollination, watering with fertilizer resumes and plants were kept well-watered. Watering was ceased 23 days after pollination (DAP) and the ears were de-husked while still attached to plants to facilitate drying and to reduce mold. Once the seed was dry enough that the endosperm could not be marred by a thumbnail (~30 DAP) the ear was harvested and dried in a seed dryer or sunny part of the greenhouse.

### Doubled Haploid

Doubled haploid (DH) lines were produced from F1 seeds of reciprocal crosses between two FFMM-AT lines, AT1 self-generation 4 (self-4, 91% Type-II callus) × AT4R self-3 (regenerated from callus of a self-2 ear that produced 66% Type-II callus). Seed from these F1 crosses were grown and crossed as a female by RWS-GFP, a haploid inducer line carrying an EGFP driven by 2x Cauliflower Mosaic Virus (CaMV) 35S promoter to facilitate identification of haploids (Röber et al., 2005, Yu and Birchler, 2016). Immature embryos were harvested 9–10 DAP for haploid doubling using two different methods described below.

#### Embryo Rescue Doubling (ERD)

For lines ATDH1 and ATDH4, embryos were plated embryo axis-side down, scutellum-side up onto a MS Rooting Medium supplemented with colchicine (Barton et al., 2014) that blocks spindle fiber formation during mitosis to cause genome doubling (Supplemental Material). Embryos were incubated in the dark at 28°C for ~24 h. All embryos were then moved to the MS Rooting Medium without colchicine and with the embryo axis-side up, scutellum-side down, to encourage the embryo to germinate. After 3–5 days incubation in the dark at 28°C, embryos were checked for GFP expression in a dark room using a NIGHTSEA BlueStar flashlight and filter glasses (NIGHTSEA, MA, USA). GFP positive (diploid) embryos were discarded and GFP negative (haploid) embryos were allowed to continue germinating. Once the coleoptile was about 2 cm long, the germinating embryos were buried upright in a sundae cup (Solo SD-12) containing the MS Rooting Medium, with just the tip of the coleoptile protruding from the MS Rooting Medium. The corresponding lids were sealed to the sundae cups and the germinating embryos were moved to a lighted biological incubator (28°C, 16 h day, 8 h night, 20-150 μmol/m^2^/s) to root. Once roots and leaves were established, the plants were transplanted to soil in a similar manner as a regenerated plantlet from transformation as described later. Doubled haploid plants showed restored fertility throughout the whole tassel and were self-pollinated to produce ears.

#### Haploid Callus Spontaneous Doubling (HCSD)

Line ATU1 was generated by plating embryos directly on Callus Development Medium (605J, Supplemental Material, Lowe et al., 2016) with the scutellum-side up and incubating in the dark at 28°C. After 3–5 days the embryos were screened for GFP expression using a NIGHTSEA Bluestar flashlight and filter glasses and GFP expressing diploid embryos were discarded. Haploid embryo callus was transferred to fresh 605J medium after 2 weeks, and incubated for additional 2 weeks. Plantlets were regenerated using Shoot Formation Medium (289O) as described (Supplemental Material, Lowe et al., 2016). Shoots were transferred to the MS Rooting Medium described above in a lighted biological incubator (28°C, 16 h day, 8 h night, 20–150 μmol/m^2^/s) until leaves and roots developed. Rooted plants were then transferred to soil as described in the transformation section below. Regenerated plantlet clones showed good fertility restoration in the tassels and were sib-crossed to produce seeds.

### Construct and *Agrobacterium* Strain

The CRISPR-Cas9 construct, pKL2013 (Supplementary Figure 1), was made by inserting a red fluorescent protein marker (mCherry) from pPT5 (Lee et al., 2019a) into A844B, which contains a gRNA targeting the maize *Glossy2* gene (Lee et al., 2019b). The mCherry cassette (CaMV 35S promoter-mCherry-Tvsp terminator) was PCR amplified from pPT5 using the primers P35S-F1 (5′-CCTTAATTAAGGGAAGACCAAAGGGCTATTGAGA-3′) and Tvsp-R1 (5′-TCCGCGATCGCCGCTTATTGCACTCCCTTTT-3′), and digested with *Pac*I and *Pvu*I (NEB, MA, USA). A844B was digested with *Pac*I and treated with thermosensitive alkaline phosphatase according to the manufacturer's instruction (Promega, WI, USA), and purified using the QIAquick PCR purification kit (Qiagen, Hilden, Germany). Ligation was performed using the T4 DNA ligase (NEB) with 50 ng of the digested A844B DNA and 15 ng the mCherry cassette in 10 μl of total reaction volume. After incubating for 15 min at 25°C, 3 μl of the ligation mixture was used for *E. coli* transformation (DH5α) using a heat shock method (Froger and Hall, 2007). Plasmid DNA was isolated using the QIAprep Spin Miniprep kit (Qiagen) and sequenced using primers pKL2013-F1 (5′-Tgaattcgacccagctttct-3′) and pKL2013-R1 (5′-tgtggaattgtgagcggata-3′) to verify the insertion of the mCherry cassette.

*Agrobacterium* strain LBA4404Thy- (Ranch et al., 2010) harboring a plasmid PHP71539 (Anand et al., 2018) was obtained from Corteva Agriscience Inc. This strain is a thymidine auxotrophic *Agrobacterium* strain that can only survive in media supplemented with thymidine (Ranch et al., 2010). The plasmid PHP71539 (Anand et al., 2018) carries extra sets of *Agrobacterium* virulence (*vir*) genes that can further enhance the *Agrobacterium*'s T-DNA transfer ability. We introduced pKL2013 into the *Agrobacterium* strain via electroporation as previously described (Mattanovich et al., 1989). After 2-day incubation at 28°C, *Agrobacterium* colonies appeared on the solid Yeast Extract Peptone medium (YP) amended with 30 mg/L gentamicin, 50 mg/L kanamycin, and 50 mg/L thymidine. Two single colonies were grown in 10 mL of liquid YP medium containing 30 mg/L gentamicin, 50 mg/L kanamycin, and 50 mg/L thymidine for 20 h at 28°C with a shaking at 200 rpm and the plasmid DNA was extracted using the QIAprep Spin Miniprep kit (Qiagen). Extracted plasmid DNA from the *Agrobacterium* cells and the original pKL2013 DNA from *E. coli* were digested with *Hin*dIII and *Pvu*I (NEB) and resolved by 1% Agarose gel electrophoresis to confirm the presence and stability of pKL2013 within LBA4404Thy- cells.

### Maize Transformation

FFMM-AT transformation experiments for delivery of the construct carrying CRISPR reagents were carried out using standard transformation protocol similar to Hi-II genotype described previously (Wang and Frame, 2004) with modifications. Briefly, the media of Wang and Frame (2004) were replaced with the following media (Supplementary Material): Liquid Infection Medium (700A), Cocultivation Medium (562V), Callus Development Medium (605J or 605T), Selection Media I and II, and Shoot Formation Medium (289O plus 3 mg/L bialaphos) per Lowe et al. (2016), Jones et al. (2019) and Masters et al. (2020).

Immature embryos of 1.5 mm average length were dissected and transferred into 700A Liquid Infection Medium in a 1.5 mL Eppendorf tube (up to 100 embryos/mL). For infection, the 700A liquid was replaced with 1 mL of *Agrobacterium* culture (OD_600_ = 0.4) that was suspended in the 700A liquid medium supplemented with 100 μM acetosyringone (AS). The embryos were infected for ~5 min at room temperature before the embryos and the liquid culture were transferred onto 562V Cocultivation Medium. Afterwards any excess *Agrobacterium* culture was removed, and the embryos were oriented scutellum-side up. The plates were wrapped with parafilm and incubated at 20°C in the dark for 3 days. After cocultivation, embryos were transferred to 605T Resting Medium and incubated for 7–10 days to begin callus initiation. Next, embryos were transferred to Selection I Medium (605J plus 3 mg/L bialaphos) and incubated at 28°C in the dark. After 2 weeks, callus pieces were transferred to Selection II Medium (605J plus 6 mg/L bialaphos) for continued selection and callus growth. Rapidly growing calli were transferred with about 6–8 calli per plate to give room for growth. Friable callus pieces were separated and put in contact with the medium. These calli were then incubated for additional 2–3 weeks in the dark at 28°C.

After selection, healthy-looking callus was evaluated with a NIGHTSEA dual fluorescent protein flashlight and RFP filter glasses. Both RFP-positive and RFP-negative healthy calli were identified and placed on Shoot Formation Medium (289O plus 3 mg/L bialaphos). These calli were incubated in the dark at 28°C for 7 days then moved to the lighted chamber described above. Within 1–2 weeks, developing shoots were transferred to MS Rooting Medium (plus 2 mg/L bialaphos). To ensure good root formation, remnant callus materials surrounding the base of the shoot were removed before the shoots were buried in MS Rooting Medium. For successful outcrossing, pollen donor seeds were planted about 9 days after the plantlets were moved to MS Rooting Medium.

Once the plants grew a total of about 7 cm of root length (either in one root or several lengths added up but not counting hair-fine roots) they were removed from medium, the roots were washed off with water, and transferred to soil (Supplementary Material). Multiple connected plantlets growing from a single piece of callus were gently teased apart for separate planting. Plantlets with one or two leaves and a good start to roots were transferred to a minimal amount of soilless substrate that was kept moist but not soaked to encourage root growth. Plantlets were covered with a humidity dome and moved to a growth chamber set to 26°C 16 h/8 h day night cycle or to the greenhouse. During the first 5–8 days in soil the plants remained covered with a humidity dome to avoid desiccation due to changing conditions.

Once established, plantlets were moved to 3 inch (7.6 cm) square nursery pots and watered with fertilizer as described above until a tassel became visible. Some plants flowered in these square nursery pots but some outgrew those pots and were moved to larger half-gallon or gallon (3.7-liter) size nursery pots before maturing. Before flowering, ear shoots were bagged similarly to seed grown plants using half shoot bags, but tassels were not covered and silks were not cut because they tended not to regrow. To pollinate, the tips of shoot bags were cut and pollen was poured onto the silks. The shoot bag was then folded over and covered by a second half shoot bag because the plants were generally not sturdy enough to hold a half tassel bag. T0 plants were also reciprocally crossed with individuals started from seed.

To prevent mold caused by unusually moist conditions within ears of regenerated plants, ears were dehusked at 11–12 DAP while remaining attached to the plant to mature, while seed-grown wild type female plants were treated per the normal protocol described above. Plants were watered until 23–25 days after pollination, then watering was ceased, and plants and seed were dried down as described above.

### Genotyping

Genomic DNA (gDNA) was extracted from maize leaf tissues using a previously published protocol (Edwards et al., 1991). About ~2 cm^2^ of fresh leaf tissue was ground in a 1.5 mL tube containing 500 μl of DNA extraction buffer (Edwards et al., 1991) with 100 μg/mL PureLink RNase A (Thermo Fisher Scientific, MA, USA), using a polypropylene homogenizing pestle attached to a cordless drill. After grinding for 10–15 s, an equal volume of chloroform (500 μL) was added to each tube and mixed thoroughly by gently inverting the tubes for 2 min. Sample tubes were centrifuged for 5 min at 21,130 × g and 300 μL of the aqueous phase was carefully transferred to a new 1.5 mL tube. To precipitate gDNA, 200 μL of isopropanol was added and thoroughly mixed by gentle inversions. gDNA was pelleted by centrifugation for 5 min and washed once with 500 μL of 80% ethanol and air dried for 10 min at room temperature. About 30–50 μL of ultrapure water was added to each tube and the gDNA concentration was quantified using the NanoDrop 1000 Spectrophotometer (Thermo Fisher Scientific, MA, USA) and adjusted to 50 ng/μL.

Genotyping analysis of *gl2* was performed by Sanger sequencing and trace data analyses using the Tracking of Indels by DEcomposition (TIDE, Brinkman et al., 2014) and Inference of CRISPR Edits (ICE, Hsiau et al., 2018). Briefly, an ~1 kb region of *gl2* was PCR amplified using the Phusion high-fidelity DNA polymerase (NEB), primers Zm-gl2-F2 and Zm-gl2-R2 (Lee et al., 2019b), and about 50 ng of gDNA. Two pairs of primers were also used to screen for the presence of the T-DNA in the transgenic plants: zCas9-F1 and zCas9-R1 (Lee et al., 2019b) for the CRISPR-Cas9 and bar-RT-F5 and bar-RT-R5 (Testroet et al., 2017) for the *bar* gene. Detailed PCR reaction composition and the thermocycling conditions were as previously reported (Testroet et al., 2017; Lee et al., 2019b). Five microliters of the PCR product was used for agarose gel electrophoresis to verify single band amplification, and amplified PCR fragments were cleaned up by treating 5 μl of PCR product with 2 μl of ExoSAP-IT reagent (Thermo Fisher Scientific) according to the manufacturer's instruction. Sanger sequencing was carried out by the DNA Facility at the Iowa State University using the oligonucleotide ZmGl2-exon2-F1 as a primer (Lee et al., 2019b). Sanger sequencing trace data were analyzed by TIDE (Brinkman et al., 2014) and ICE (Hsiau et al., 2018) using the default settings and the wild type FFMM-A *Gl2* sequencing trace file as a control.

### Phenotyping and Mutant Inheritance Analysis

T0 plantlets were screened for loss-of-function *gl2* mutants by misting seedling leaves with water once the plants had acclimatized to the low-humidity conditions of the growth chamber, as well as by PCR as described above. T1 seeds were screened by germinating in vermiculite then screening roots for mCherry expression using a NIGHTSEA dual fluorescent protein flashlight and filter glasses designed to visualize RFP. Once seedling leaves emerged, leaves were misted with water to identify loss-of-function *gl2* mutants.

For T1 seed of marginal quality and likely incapable of germinating properly in vermiculite, an embryo rescuing method was used as described (Martinez and Wang, 2009). The surface sterilized mature embryos were placed embryo-axis-side up on MS medium containing 100 mg/L of benomyl to germinate. Rooted plants were moved to soil using the same method as described for a regenerated plantlet from transformation described above with the exception plants were moved to a 1 gallon (3.7-liter) pot and treated similarly to, and resembled, a seed grown plant once established.

## Results

### Generation of Embryogenic Callus Producing FFMM Lines

**Figure 1:**
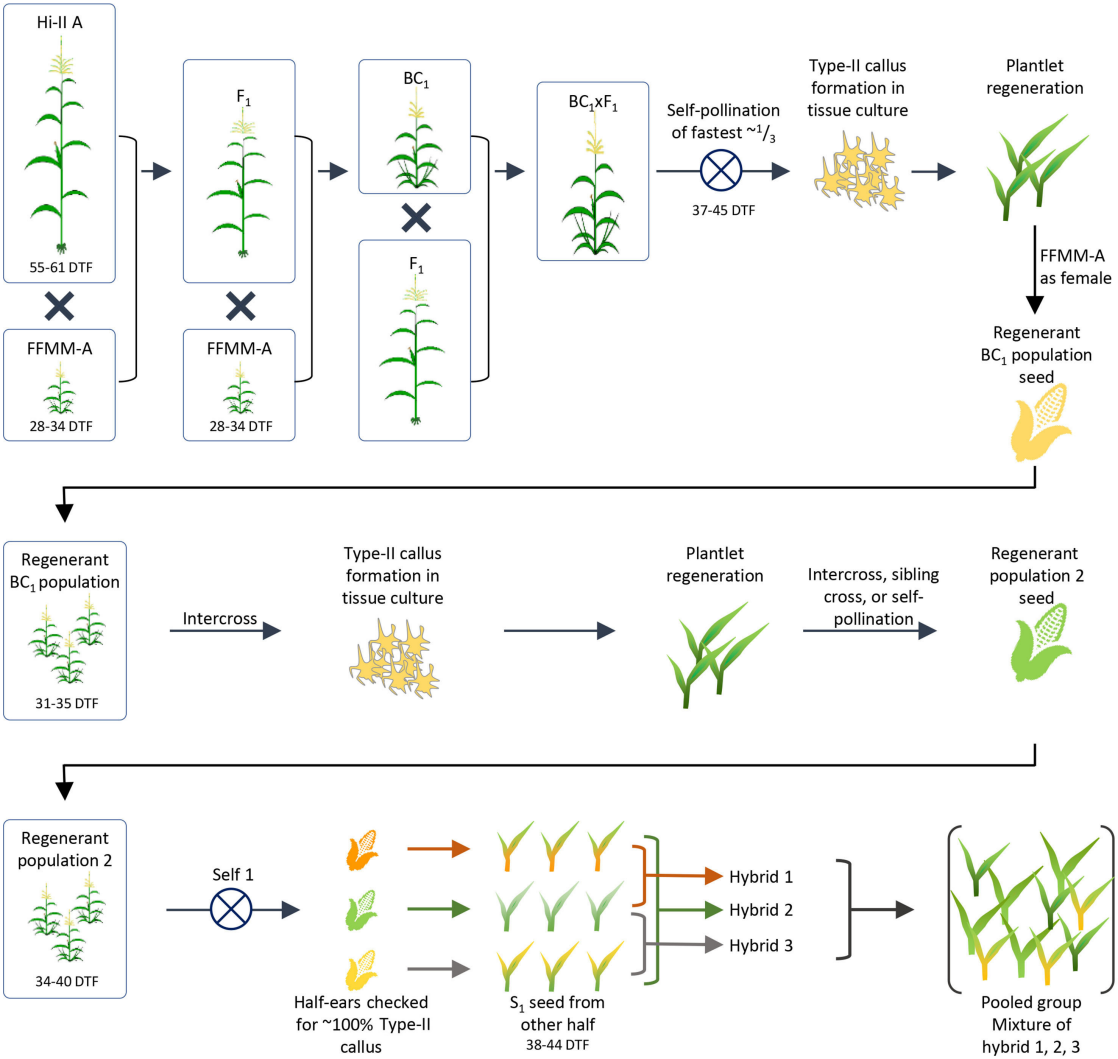
Generation of initial FFMM-AT population. The breeding process used to create a population of plants with FFMM characteristics and the ability to form Type-II callus in tissue culture. From an initial F1 × BC1 crossing, callus was induced and regenerated plants were backcrossed as males to FFMM-A. The population was passed through tissue culture again to select for formation of Type-II callus, and regenerated plants formed a second population. Half-ears of selfed plants were checked for ~100% Type-II callus responses and presumed fixation of the trait. Plants derived from the seed of the other half of ears resembled FFMM-A but took too long to flower so they were intercrossed to form a final population from which fast-flowering lines could be selected. DTF, days to flowering.

Early efforts in attempting to transform FFMM–A and –B lines were unsuccessful in our hands because they are unable to produce embryogenic callus using standard conventional tissue culture and transformation protocols. To breed transformable FFMM lines, we performed a series of crosses and backcrosses by hybridizing the FFMM-A line with Hi-II Parent A (Hi-II A) (Figure 1). Hi-II is a transformable genotype; its immature embryos can readily produce friable, embryogenic callus culture (Armstrong et al., 1991). The goal was an introgression of competency to form embryogenic callus in tissue culture from Hi-II A into the FFMM-A genetic background to generate a transformable line FFMM-AT.

An initial F1 hybrid between Hi-II A and FFMM-A then a backcross 1 (BC1) to FFMM-A were produced. These materials were crossed to generate F1 × BC1 seed, which was grown to maturity. The fastest flowering plants were selfed (37–45 days after planting, representing about 1/3 of the total plants). Embryos were harvested 9 DAP and directly placed onto N6 callus induction medium (Wang and Frame, 2004) to evaluate their ability to produce Type-II callus. Type-II callus was produced by only 30 independent embryos and of those 15 callus events were able to form plantlets in regeneration media as described (Wang and Frame, 2004).

Plants resulting from nine separate callus events were back-crossed as a male to FFMM-A ear donors creating a ~BC1.5. Grown from seed, these ~BC1.5 plants strongly resembled FFMM-A in both plant architecture and flowering time (31–34 days). These backcrosses were intercrossed in as many unique combinations as possible, generating 18 ears from which embryos were extracted and taken through tissue culture as described above. The regenerated plants were grown in an isolated greenhouse and pollinated by pooling pollen then using it to fertilize open silks, creating a population of self-pollinated, sib-pollinated, and intercrossed seeds (Figure 1).

**Figure 2:**
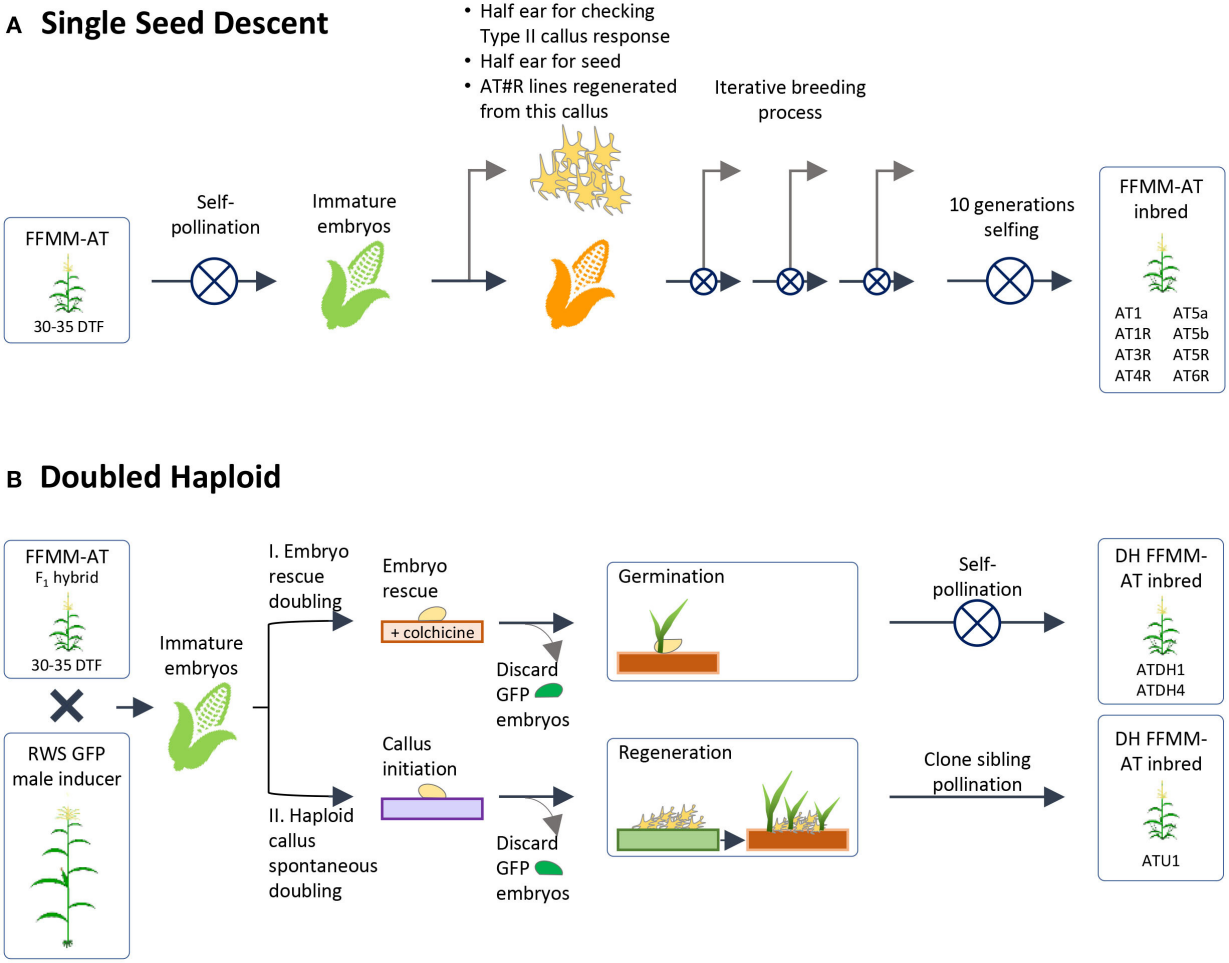
Generation of homozygous lines. This figure depicts the two methods by which homozygous lines were produced from a heterozygous population selected for callus formation and regeneration, fast-flowering, and a Mini-Maize plant architecture. **(A)** Single Seed Descent method of inbreeding with further selection for callus formation and desired plant phenotype; **(B)** Doubled haploid methods. Starting FFMM-AT material was an F1 hybrid of lines from **(A)** that produced high rates of Type-II callus formation when half ears were checked and/or regenerated. DTF, days to flowering.

Seeds from the second round of tissue culture and regeneration were grown to maturity and self-pollinated (Figure 1). The top half of ears were harvested 9–11 DAP when embryos were ~1.5 mm long, and embryos were cultured on N6 medium to evaluate Type-II callus generation frequency. Three half-ears yielded ~100% Type-II callus response and seeds in the remaining lower half of the ears were grown to maturity. Plants grown from these seeds resembled FFMM-A, but were slow to flower (38+ days vs. 28–34 days for FFMM-A), so these 3 lines were intercrossed once again. Seed from nine separate ears was then initiated into single seed descent inbreeding with selection for fast-flowering, good seed set, and ample pollen shed (Figure 2A). These lines were designated as FFMM-AT lines.

**Figure 3:**
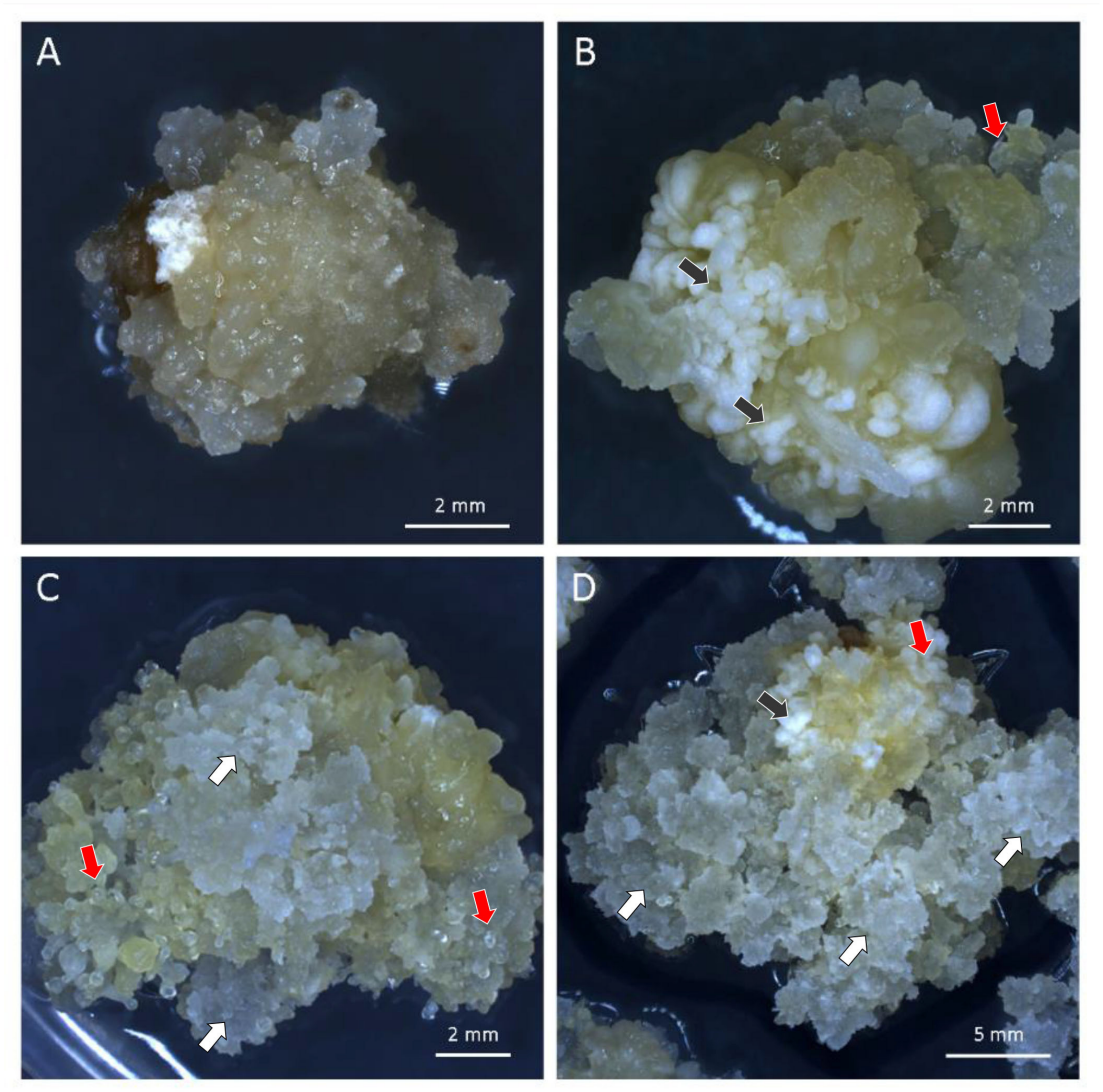
Different types of embryogenic callus. **(A)** Non-embryogenic callus of FFMM-A; **(B)** Embryogenic callus of FFMM-ATU1; **(C,D)** Embryogenic callus of FFMM-AT6R. Black arrows, Type-I compact callus; White arrows, Type-II friable callus; Red arrows, Type-1.5 callus. All three regenerable types were produced from 9 DAP immature embryos and cultured on 605 J medium for 26 days.

Embryogenic Type-II callus response was checked again at self-generation 2 (self-2) for one batch and self-3 for a second batch. Two independent lines, FFMM-AT1 and FFMM-AT5, which had close to 100% callus response, continued through single seed descent. These produced lines FFMM-AT1, FFMM-AT5a, and FFMM-AT5b. In addition, these three lines and three additional lines (FFMM-AT3, AT4, AT6) with reduced Type-II callus initiation frequencies were regenerated from callus once again before resuming selfing (Figure 2A). This produced lines FFMM-AT1R, AT3R, AT4R, AT5R, and AT6R. After self-hybridization for 10 generations, eight independent FFMM-AT inbred lines with the ability to produce embryogenic callus were established (Figures 2A, 3). Compared to genotypes from the early breeding cycle, the embryogenic callus morphology produced from these finished lines is not as friable as the Type-II callus (Figure 3D), but rather somewhat resemble embryogenic Type-I callus type (Figures 3B,C), so we call it Type-1.5.

**Figure 4:**
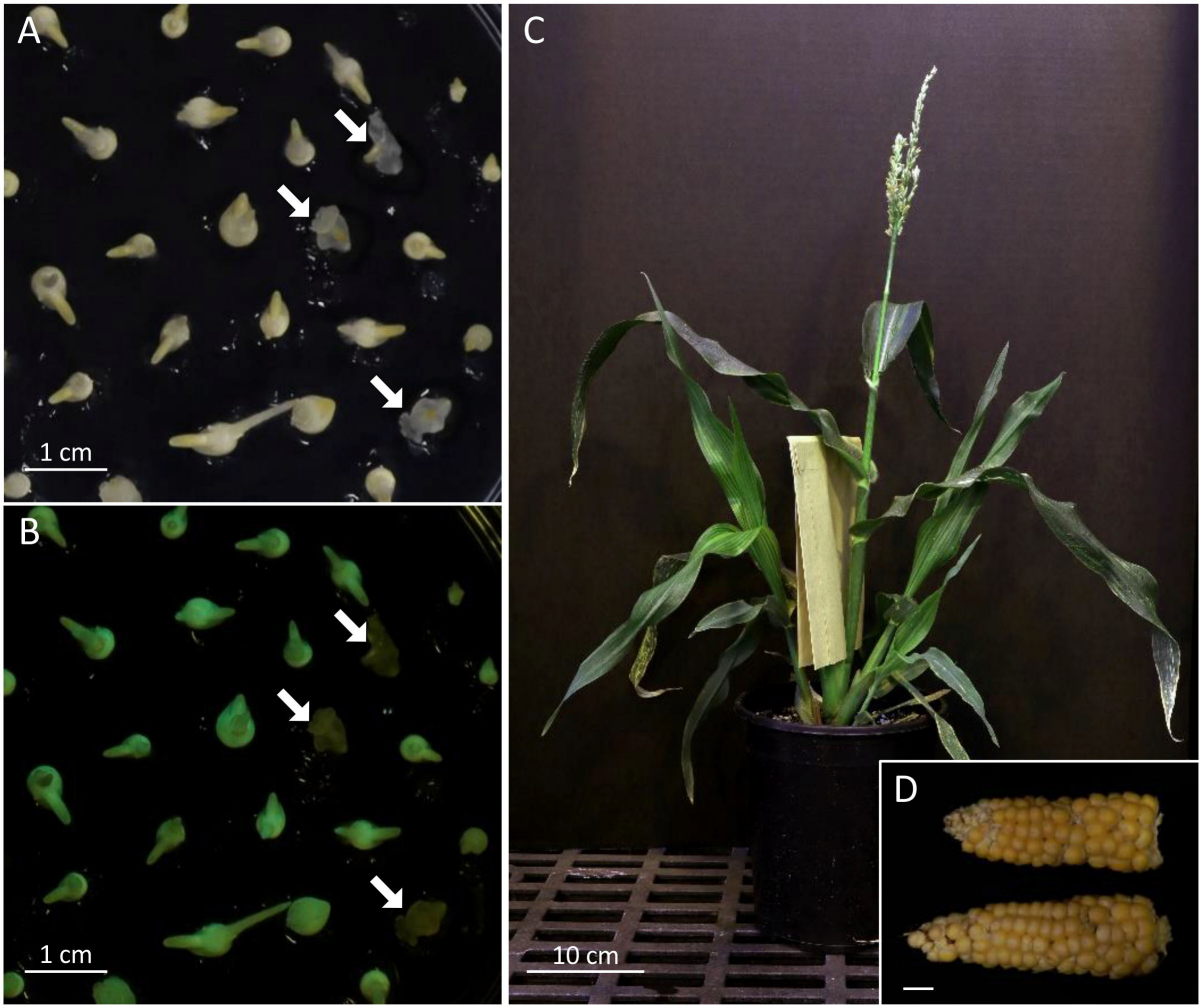
FFMM-AT. Screening of haploid embryos under bright field **(A)** and NIGHTSEA flashlight with GFP filter glasses **(B)**. White arrows point to non-GFP-expressing haploid embryos. **(C)** A mature FFMM-AT plant; **(D)** Mature ears of AT6R; bar scale, 1 cm.

As an alternative to self-hybridization to reach homozygosity, we also attempted a faster breeding process using Doubled Haploid (DH) technology (Figure 2B). The F1 hybrid seeds from a cross between AT1 (self-4, 91% Type-II callus) and AT4R (self-3 regenerated from an ear with 66% Type-II callus) were grown and crossed as a female by RWS-GFP, a haploid inducer line carrying a green fluorescent marker gene (GFP) to facilitate identification of haploids (Röber et al., 2005; Yu and Birchler, 2016). Under the NIGHTSEA BlueStar flashlight, the immature diploid embryos were fluorescent due to the presence of the paternal *gfp* transgene, and thus were discarded (Figure 2B). Non-fluorescent embryos (Figures 4A,B) were treated either by embryo rescue doubling (ERD, Barton et al., 2014) or haploid callus spontaneous doubling (HCSD) method. Three doubled haploid FFMM-AT lines, ATDH1, ATDH4, and ATU1 were generated (Figure 2B).

### *Agrobacterium*-Mediated Targeted Mutagenesis in FFMM-AT

**Figure 5:**
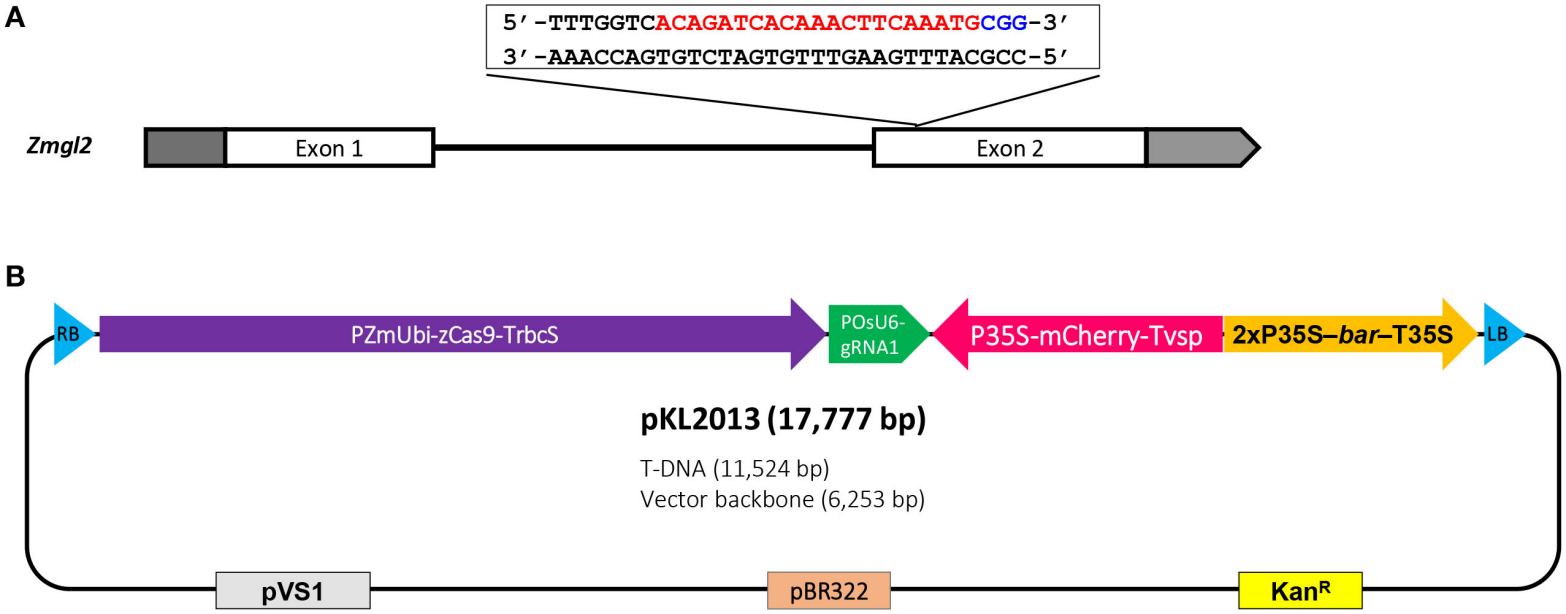
Schematic illustration of maize *glossy2* (*gl2*) gene target sequence and CRISPR-Cas9 construct for *Agrobacterium*-mediated transformation. **(A)** Maize *Gl2* has two exons and the target sequence is located near the 5′ end of the second exon. The protospacer (red) and PAM (blue) are indicated; **(B)** The CRISPR-Cas9 construct, pKL2013, has an 11.5 kb T-DNA which includes maize-codon-optimized SpCas9 driven by maize ubiquitin promoter, sgRNA1 cassette driven by rice U6 promoter, mCherry cassette driven by CaMV 35S promoter, and *bar* selectable marker driven by CAMV 35S promoter. RB, T-DNA right border; PZmUbi-zCas9-TrbcS, maize ubiquitin promoter driving maize-codon-optimized SpCas9 (zCas9) with ribulose-1,5-bisphosphate carboxylase/oxygenase small subunit 2b terminator; POsU6-gRNA1, rice U6 promoter driving sgRNA1 with rice-U6-2 terminator; P35S-mCherry-Tvsp, CaMV 35S promoter driving mCherry with soybean vegetative storage protein terminator; 2xP35S-bar-T35S, CaMV 35S promoter driving phosphinothricin N-acetyltransferase (*bar*) with CaMV 35S terminator; LB, T-DNA left border; pVS1, origin of replication from the plasmid pVS1; pBR322, origin of replication from the plasmid pBR322; Kan^R^, aminoglycoside phosphotransferase gene providing resistance to kanamycin.

Table 1 summarizes the transformation experiments carried out on 10 out of 11 advanced FFMM-AT genotypes that were generated from either > 7 generations of self-pollination or doubled haploid treatments. The CRISPR construct pKL2013 (Figure 5, Supplementary Figure 1) has an 11.5 kb T-DNA that carries a mCherry marker gene for visible selection, *bar* gene for plant selection, and SpCas9 and sgRNA for targeted mutagenesis of *Gl2*. The gene product of *Gl2* is responsible for the formation of a hydrophobic waxy cuticle layer in juvenile leaf tissues, and the knockout mutants can be easily identified by misting water on the young leaf surface (Bianchi et al., 1975). Water will roll off wild type *Gl2* plants but droplets will adhere to the *gl2* homozygous or biallelic knockout individuals.

**Table 1:** Summary of FFMM-AT transformation experiments.

**Exp ID**	**Genotype name**	**Self-generation**	**# of ear**	**# embs infected**	**# RFP+ callus**	**# event w/ shoot**	**# event** **rooted**	**# event** **to gh**	**# event** **to seed**	**% TF**	**Avg**	**Std**
1	AT1	9	1	97	7	5	5	5	1	1.0%	**1.1%**	0.1%
2	AT1	10	2	81	4	2	2	2	1	1.2%		
3	AT1R	8	3	125	1	1	1	1	0	0.0%	**0.0%**	0.0%
4	AT1R	9	2	153	3	1	0	0	0	0.0%		
5	AT3R	7	2	145	1	5	5	3	2	1.4%	**N/A**	N/A
6	AT4R	8	2	156	14	9	9	5	4	2.6%	**1.5%**	1.5%
7	AT4R	10	4	241	2	1	1	1	1	0.4%		
8	AT5b	8	2	124	1	2	2	1	1	0.8%	**N/A**	N/A
9	AT5R	7	1	113	15	9	9	9	2	1.8%	**N/A**	N/A
10	AT6R	7	1	72	35	28	26	22	4	5.6%	**11.3%**	8.1%
11	AT6R	8	2	41	9	15	7	7	7	17.1%		
12	ATDH1	DH	2	79	5	2	2	2	2	2.5%	**3.4%**	1.3%
13	ATDH1	DH	2	23	2	1	1	1	1	4.3%		
14	ATDH4	DH	1	42	1	1	1	1	0	0.0%	**0.9%**	1.2%
15	ATDH4	DH	1	58	1	1	1	1	1	1.7%		
16	ATU1	DH	1	49	13	10	9	8	5	10.2%	**7.0%**	4.5%
17	ATU1	DH	2	103	12	6	6	6	4	3.9%		
**Total**			**31**	**1702**	**126**	**99**	**87**	**75**	**36**			

*DH, germplasm generated using Doubled Haploid technology*.

# *RFP+ callus, putative transgenic callus with red fluorescence*.

*TF, transformation frequency (# event to seed/#embryos infected × 100)*.

*Avg, average transformation frequency; Std, standard deviation; N/A, not applicable*.

To enhance transformation, the construct is mobilized into an *Agrobacterium* thymidine auxotrophic strain LBA4404Thy- harboring a helper plasmid PHP71539 that carries an extra copy of virulence (*vir*) genes from the pTiBo542 plasmid (Anand et al., 2018). Extra copies of *vir* genes in an *Agrobacterium* strain have been shown to be effective in enhancing transformation frequency (Komari, 1990; Ishida et al., 1996; Anand et al., 2018). The Thy- strain was used to minimize *Agrobacterium* carryover during transformation process because this strain cannot survive on media without the addition of thymidine (Ranch et al., 2010). Over 1,700 immature embryos representing 31 ears were dissected and infected in a total of 17 individual experiments.

As shown in Table 1, immature embryos of all 10 FFMM-AT genotypes were capable of producing Type-1.5 embryogenic callus on media described in this work, with FFMM-ATU1 and FFMM-AT6R occurring at high frequencies (over 70%). FFMM-AT6R often produces a Type-II callus response in addition to Type-1.5 as can be seen in Figure 3D. After infection and co-cultivation, bialaphos-resistant callus pieces were monitored for the red fluorescent protein (RFP) mCherry expression throughout the selection stage. A total of 126 RFP-positive callus pieces (out of 1,702 infected embryos) were scored; 99 of the bialaphos-resistant callus pieces produced shoots and 87 of them made roots. Among 75 events transplanted to the soil, about half of them (36 events) produced seed. The transformation frequency (TF), defined as the number of transgenic events that produced T1 seeds per 100 embryos infected, ranged from 0.0% for genotype AT1R to 17.1% for AT6R. Among seven single seed descent, self-pollinated FFMM-AT genotypes tested, AT6R produced the highest TF with an average of 11.3 ± 8.1% (mean ± *SD*). Among the three doubled haploid lines, genotype ATU1 produced a high TF with an average of 7.0 ± 4.5% (Table 1).

### T0 and T1 Analysis

Phenotyping and genotyping were performed for all T0 plants. The mCherry expression in root tissue was examined by a hand-held flashlight device. Leaf materials of T0 plantlets were analyzed by PCR for the presence of the *bar* and Cas9 genes. Then the target gene *gl2* was analyzed by Sanger sequencing and trace file analyses using the TIDE (Brinkman et al., 2014) and ICE (Hsiau et al., 2018) analyses.

Table 2 summarizes the T0 mutant genotypes. Out of 86 T0 plants sequenced, 68 plants carried mutations in the *gl2* gene, giving a mutagenesis frequency of 79%. Among the 18 plants with the wild-type genotype, 13 of them were Cas9-positive plants. It is possible that the Cas9 gene expression was silenced in these lines.

**Table 2:** Summary of T0 mutant genotypes^†^.

	**# plants**	**% T0 mutant**
Homozygous	20	23.3%
Biallelic	37	43.0%
Heterozygous	0	0.0%
Mosaic	11	12.8%
Wild type^*^	18	20.9%
Total analyzed	**86**	**100.0%**

† *Homozygous, one mutant sequence without wild type allele; Biallelic, two different mutant sequences; Heterozygous, wild type sequence and one mutant sequence; Mosaic, three or more mutant sequences in a single plant*.

* *Among the 18 WT plants, 13 were Cas9 positive*.

**Figure 6:**
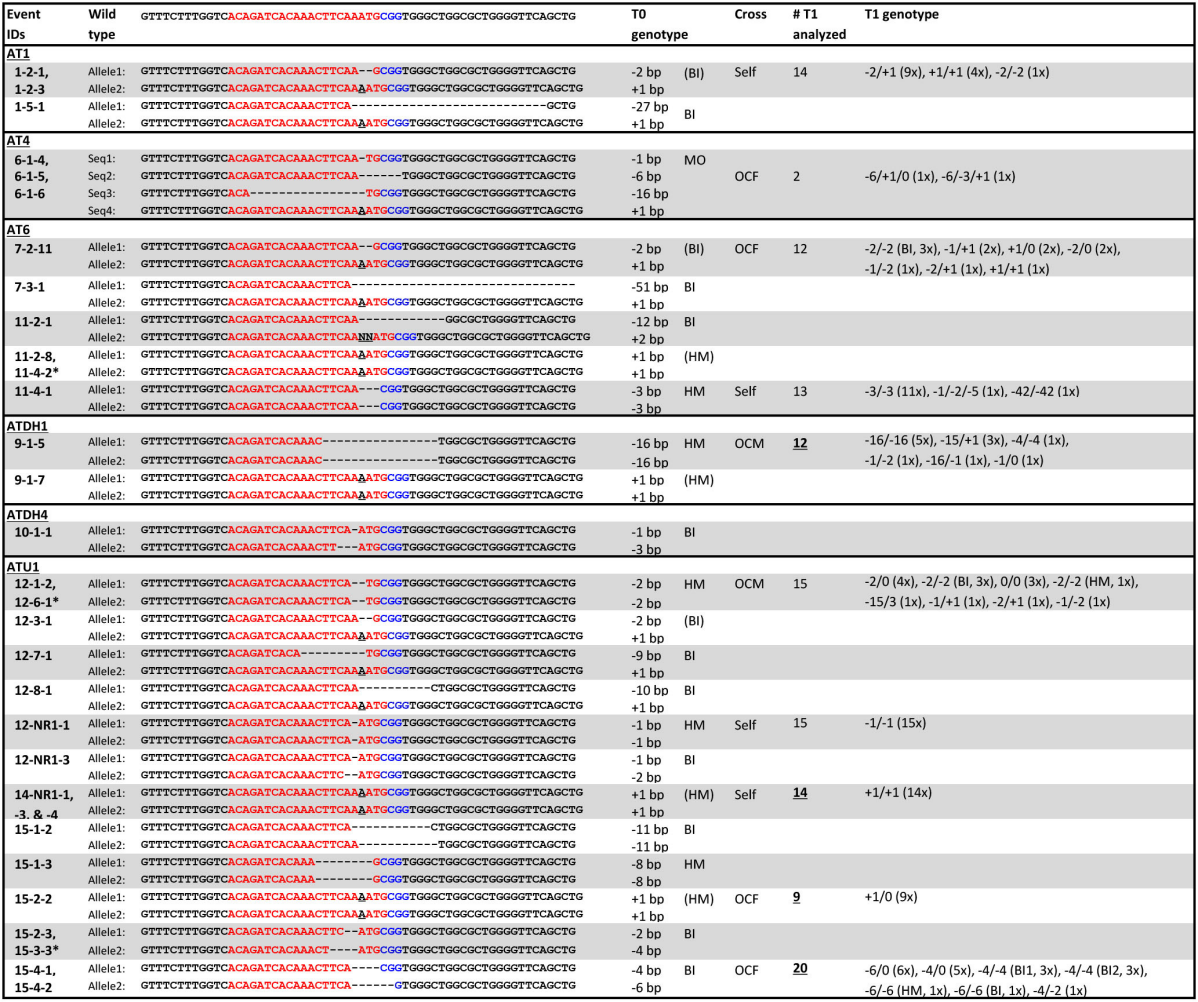
Genotypes of selected T0 and T1 mutants. Red letters, target sequences in *Gl2* exon 2; Blue letters, PAM sequences; Black letter with underscore, insertion mutations; AT1, AT4R, AT6R, ATDH1, ATDH4, and ATU1, FFMM-AT genotypes; Event marked with star, separate event that had the same mutation pattern; BI, biallelic; HM, homozygous; MO, mosaic; (BI) and (HM), events with same mutation pattern in more than one sampled T0 plant; Self, self-pollination; OCF and OCM, an outcross with the T0 plant used as a female or male, respectively; Underlined numbers, T1 progenies of T0 plants of which their sibling T0 plants from the same transgenic event were sequenced; T1 genotypes, numbers in parentheses indicate the numbers of plants from the event that have the same mutation pattern.

The *gl2* target sequences of selected T0 mutant lines are listed in Figure 6. As can be seen, most mutants have insertions or deletions (indels) near the PAM sequence. Some sibling plants derived from the same transgenic callus lines have the same mutation patterns, such as lines 1-2-1 and 1-2-3; 6-1-4, 6-1-5 and 6-1-6; 14-NR1-1, 14-NR1-3 and 14-NR1-4; as well as 15-4-1 and 15-4-2. In these events, targeted mutagenesis likely occurred at an early stage before the callus induction. Sometimes, plants produced from different callus lines can have the same mutation patterns, such as lines 11-2-8 and 11-4-2; 12-1-2 and 12-6-1; and 15-2-3 and 15-3-3. Two mutation patterns,−2/+1 (biallelic) and +1/+1 (homozygous), were prevalent and can be detected in a number of T0 mutants that were generated in different and separate infection experiments. Four plants (1-2-1, 1-2-3, 7-2-11, and 12-3-1) have the biallelic−2/+1 genotype and seven plants (11-2-8, 11-4-2, 9-1-7, 14-NR1-1,−3,−4 and 15-2-2) have the homozygous +1/+1 genotype (Table 2).

On the other hand, sibling plants derived from the same transgenic callus can often carry different mutation patterns (Table 2). For example, 11-2-1 and 11-2-8 were siblings from the same callus event. They have different mutant patterns; 11-2-1 is biallelic (-12/+2) and 11-2-8 is homozygous (+1/+1). Likewise, 9-1-5 and 9-1-7, 12-NR1-1 and 12-NR1-3, 15-1-2 and 15-1-3, 15-2-2 and 15-2-3 were all sibling plants with each other, but carried different mutant genotypes. This phenomenon has been reported in previous work (Char et al., 2017; Banakar et al., 2019; Lee et al., 2019b) and suggests that the *gl2* mutation might have occurred after initial cell divisions of the transformed cells. If the CRISPR reagents were not expressed fully at the early stage (single cell) of transformation, chimeric callus culture can generate multiple plants with different mutation patterns, even though they are all derived from a single transgenic event. There is also the possibility that multiple transgenic events were produced from a single embryo and were both represented during callus formation and selection.

**Figure 7:**
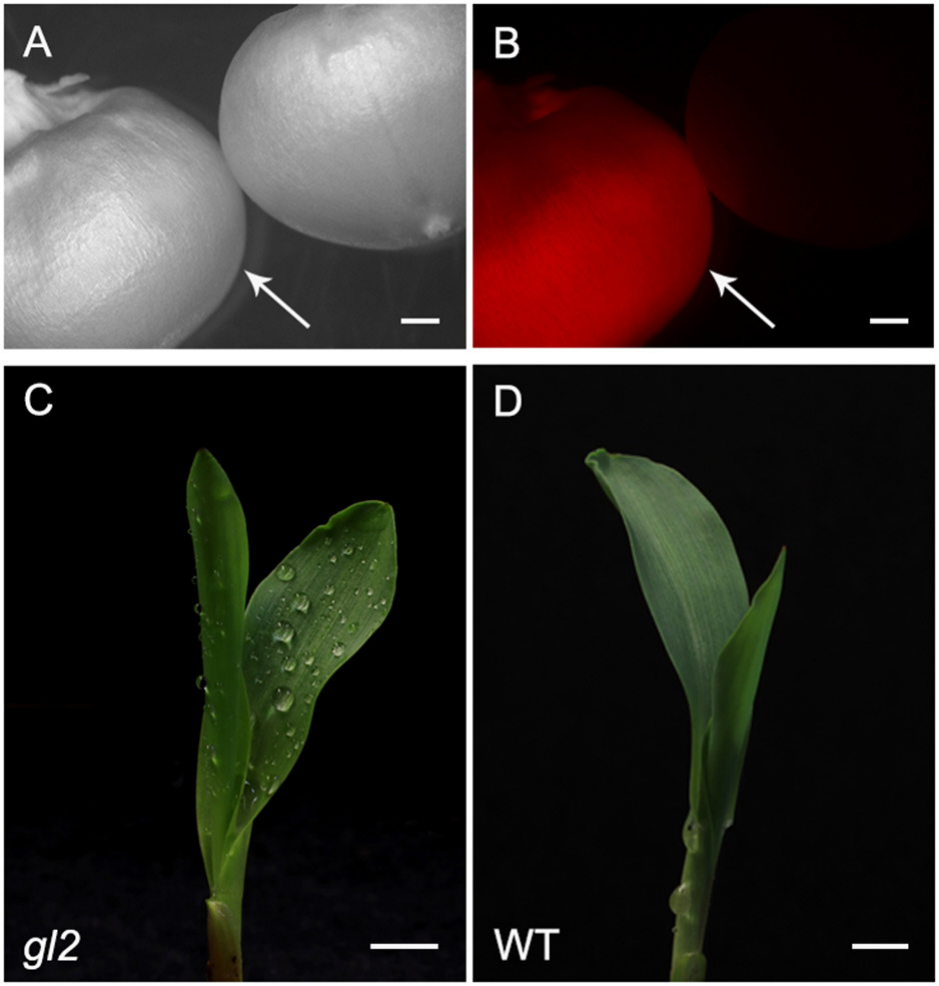
Representative phenotypes of T1 transgenic lines. T1 progeny seed of RFP expressing (left, indicated by an arrow) and non-expressing segregant (right) under bright light **(A)** and RFP channel **(B)**. The phenotype of *gl2* loss-of-function mutant **(C)** and wild type **(D)**. Dull surface of juvenile leaf of *gl2* mutant retains sprayed water drops. Bar scale, 5 mm.

Selected T0 mutant lines were either self-pollinated or out-crossed to FFMM-AT to produce T1 seeds. T1 seeds showed segregation of mCherry expressing transgene (Figures 7A,B) as well as *gl2* mutant phenotype (Figures 7C,D). T1 genotyping was carried out on progenies of either direct descendants or sibling plants from sequenced T0 mutant plants.

T1 seedlings from four self-pollinated lines (1-2-1, 11-4-1, 12-NR1-1, and 14-NR1-1) show parental genotypes, although lines 1-2-1 and 11-4-1 also produced some T1 seedlings with mutation patterns that were not detected in their T0 parental plants (Figure 6). Four out-crossed as female (OCF) lines and two out-crossed as male (OCM) lines gave various mutation patterns; some inherited the parent mutations but some did not. Out of the 10 T1 progenies analyzed, five of them (9-1-5, 14-NR1-1, 15-1-2, 15-2-2, and 15-4-1) were not direct descendants from their T0 sequenced parents, but rather progenies from their sibling plants derived from the same transgenic callus events. Some of them such as line 14-NR1-1 had exactly the same mutation pattern as its parent; other lines carried different patterns compared to their T0 sequenced counterparts.

## Discussion

Here we describe the successful development, transformation, and gene editing of new Fast-Flowering Mini-Maize lines, FFMM-AT. FFMM-AT lines reliably produce embryos ~6 weeks after seed germination. The transformation process takes about 8–9 weeks, followed by about 3 weeks of regenerated plant growth in soil until crossing. Seed formation and maturation takes an additional 4 weeks. This gives a seed-to-T1 seed time of ~5.5 months. Compared to transformation of over 9 months for Hi-II (Frame et al., 2015) and over 11 months for the B104 inbred (Raji et al., 2018), this is much shorter due largely to the faster generation time of FFMM on the pre-transformation material generation and post-regeneration stages. The callus selection step of FFMM-AT is also shorter than B104 and less labor intensive.

The challenges in using FFMM compared to standard maize lines are largely in adapting to care protocols that are specific to FFMM genotypes. It is important to grow FFMM in small containers such as 1-gallon (3.7-liter) pots [6.75 inches (17 cm) depth × 6.25 inches (16 cm) diameter] and avoid overwatering. Because of the rapid life cycle, stressed FFMM plants are unlikely to have enough time to recover and produce healthy pollen or ears. Seed production of 50 FFMM-AT transgenic plants generated in this study ranged from 5 to 199 with an average of 76 seeds per cob (Figure 4D), comparable to what has been observed in the original FFMM-A line (McCaw et al., 2016).

To date, successful maize transformation on a specific genotype often relies on the ability to produce embryogenic callus of the said genotype. The original FFMM lines lack this ability when using the conventional protocols and media regimes. Successful introgresssion of the ability to form Type-II callus into B73 has been reported, and the regions of the A188 genome that could be important to this ability have been identified (Armstrong et al., 1992; Lowe et al., 2006). Because of the possible existence of unknown repressors and other genetic factors, however, we decided to pursue the classic, albeit time consuming, breeding method and selected for a callus development phenotype rather than employing the marker-assisted breeding technology.

Once Type-II callus formation ability was introgressed into a background resembling FFMM, we produced homozygous lines by both inbreeding and doubled haploid approaches to achieve a uniform genetic background. Two doubled haploid methods were employed. In the ERD method invented by Barton et al. (2014), immature haploid embryos were cultured on colchicine-containing medium. In the HCSD method described in this work, immature haploid embryos were allowed to form haploid callus and undergo spontaneous chromosomal doubling without any doubling agent. Diploid plants generated from both methods showed good fertility restoration in the whole tassel, rather than sectors or branches as seen in traditional chromosome doubling methods that were used to treat haploid seedlings (Kato and Geiger, 2002; Vanous et al., 2017). The HCSD method may be particularly useful for generation of new lines capable of forming embryogenic callus because its success is determined by presence of callus formation ability in the haploid genome. Diploid homozygous lines are much more vigorous than their haploid counterpart; spontaneous genome doubling of haploid callus cells to produce diploid cells should increase the vigor of the callus and the increased growth rate can be selected for these events.

The FFMM-AT lines generated in this work demonstrated different tissue culture responses and transformation frequencies. Each line has unique characteristics to its phenotype. Line FFMM-AT6R appears to have robust performance in tissue culture and transformation capability in our study. When cultured on a 605J based medium this inbred line shows vigorous callus formation for both Type-1.5 and Type-II callus. It also has a high rate of infection by *Agrobacterium*, giving 44/113 embryos producing RFP-positive callus and 33/113 embryos producing shoots after selection.

FFMM-AT6R would be our preferred transformable line due to its robust response in callus, though it does have some differences compared to FFMM-A. FFMM-AT6R has a slightly more elongated stature that facilitates ear shoot bagging. The plant tends to produce a single-branched tassel or two tassel branches as compared to three or more in other FFMM lines; however, its pollen shed and nicking are still sufficient to pollinate the ear well. The ear tends to be shortened compared to FFMM-A, and often masculinized at the tip. It also tends to have larger kernels that are more disordered in kernel row ordering and kernel orientation. The larger kernels usually take longer to dry down before harvest. The plants have a slightly longer time to flowering, which makes FFMM-AT6R closer to seed-to-seed in 65–70 days, instead of 60 days for FFMM-A.

Several FFMM-AT lines were promising in early stages of breeding. For example, lines AT1 and AT4R produced plants that were subjectively superior to FFMM-A in plant architecture and ear size, while maintaining fast-flowering and fast seed maturity. At self-4, line AT1 showed strong ability in producing Type-II callus (over 90% from 1.2 to 1.8 mm ideal sized embryos) on the standard N6 medium used for Hi-II (Wang and Frame, 2004). At self-5 and self-6, line AT1 performed poorly on N6 medium, but performed well on 605J medium. Interestingly, at later selfed generations line AT1 performed poorly on both N6 and 605J media losing the ability to form embryogenic callus. This sudden change of tissue culture responses is puzzling. It is possible that one or a few alleles that were responsible for Type-II response were lost during the self-hybridization process.

The regeneration process of FFMM-AT is a key to success. The conventional maize transformation process for regular-sized genotypes such as Hi-II or B104 focused on producing a well-established plant with three to five leaves and a substantial root structure in tissue culture before moving to soil. Early attempts at regeneration of FFMM-AT lines by methods successful for Hi-II yielded plants with a small, fertile tassel, but they were unable to produce ears reliably. It was observed that when producing transgenic FFMM-AT plants, it is important that the regenerated plantlets to be moved from culture media to soil much earlier. Regenerated FFMM-AT plants that closely resembled a freshly sprouted seedling, with just one or two leaves and established but short roots (just >7 cm total length of thick, not hair-fine), produced much more vigorous plants in soil in our hands. These plants were much more likely to produce ears that formed fertile tassels and produce viable seeds. Regenerated plants were often smaller than seed-grown FFMM-AT plants and produced excessive moisture within the ear. This moisture necessitates the dehusking of the ears around 11–12 DAP while remaining attached to the plant to prevent fungal growth but retain development by nourishment from the plant.

CRISPR-mediated targeted mutagenesis was efficient in FFMM-AT lines with a 79% mutation rate in T0 plants (Table 2). Observed mutation patterns were mostly short indels, similar to the maize B104 *gl2* mutant events transformed with construct A844B, which carried the same SpCas9-gRNA cassettes used in pKL2013 (Lee et al., 2019b). The combined frequencies of homozygous and biallelic mutants were comparable with 63% in B104 (Table 1 in Lee et al., 2019b) and 66% in FFMM-AT. Interestingly, while no deletions larger than 7 bp were observed in B104 T0 plants, some FFMM-AT T0 lines showed large deletions over 10 bp (Figure 5). Another difference was the frequencies of heterozygous or mosaic mutants: while B104 showed a high frequency of heterozygous mutants with 37% (Table 1 in Lee et al., 2019b), FFMM-AT T0 lines had no heterozygous mutants but had mosaic mutants in 11.8% of T0 plants. It is not clear if these differences reflect any genetic divergence in the FFMM-AT lines, but our data indicate that gene editing technologies can be used efficiently in FFMM-AT lines.

FFMM-AT has obvious and direct application to maize genomics studies, especially for large-scale indoor research. FFMM-AT provides unique benefits as a model organism by shortening the timeline and reducing the greenhouse space required for experiments. A full size FFMM plant can be grown in an inexpensive growth chamber that is too small for standard maize lines (Tran and Braun, 2017). Therefore, use of FFMM for research can potentially avoid the need for a greenhouse to grow maize. In 2018, a miniature rice germplasm, Xiaowei, was reported for large-scale indoor genomic research for rice (Hu et al., 2018). Compared to a typical rice variety Nipponbare (60 cm in height and 73 days-to-heading), Xiaowei measures 11.6 cm in height and 46 days-to heading. A regular maize genotype Hi-II is nearly 2 m in height and its seed-to-seed time is about 120 days. The FFMM-AT reported in this study, measures ~90 cm in height (Figure 4C) and ~65 days from seed to seed.

While FFMM will not be suitable for analyzing all gene functions, it can be useful for studying genes and pathways where a specific genetic background is not required. Coupled with CRISPR-Cas genome editing tools, it can accelerate maize genomic research. Moreover, pollen of FFMM can be potentially useful in small grain genomic research. Recently, Kelliher et al. (2019) has shown that transgenic maize pollen expressing CRISPR reagents could be used to generate haploid wheat with expected mutations in the targeted wheat gene. It is conceivable that FFMM-AT, the transformable, short stature and life cycle maize, can be an appealing tool for CRISPR-mediated mutagenesis in wheat and other small grain crops. In summary, with reduced space requirements and generation time, adding competency for genetic transformation completes FFMM-AT as an open source tool for maize genomic research.

## Data Availability Statement

The raw data supporting the conclusions of this article will be made available by the authors, without undue reservation. FFMM-AT seeds can be obtained from James A Birchler, birchlerj@missouri.edu.

## Author Contributions

MM and KW designed and oversaw the entire project. MM performed plant breeding, tissue culture evaluation, and data collection. KL designed/built the construct and performed molecular analysis. MM, MK, and JZ performed maize transformation. JZ contributed to regeneration protocol design. MM and MA took care greenhouse plants. MM, MK, and MA performed progeny phenotyping and figure preparation. JB contributed to breeding design and planning. MM, KW, and KL performed data analysis and prepared the manuscript. All authors contributed to discussion and revision of the manuscript.

## Conflict of Interest

The authors declare that the research was conducted in the absence of any commercial or financial relationships that could be construed as a potential conflict of interest.
