# Supplementary material for: Development of a Transformable Fast-Flowering Mini-Maize as a Tool for Maize Gene Editing
Source: Front Genome Ed. 2021 Jan 11;2:622227. doi: 10.3389/fgeed.2020.622227 (PMC8525386; doi:10.3389/fgeed.2020.622227)
Supplement: Supplementary file 1 [file Data_Sheet_1.docx]

# Supplementary Materials

Development of a Transformable Fast-Flowering Mini-Maize as a Toolkit for Maize Gene Editing

Morgan McCaw^1,2^, Keunsub Lee^1,2^, Minjeong Kang^1,2,3^, Jacob D. Zobrist^1,2,4^, Mercy K. Azanu^1,2,3^, James A. Birchler^2,5^, Kan Wang^1,2*^

^1^Department of Agronomy, Iowa State University, Ames, Iowa, USA

^2^Crop Bioengineering Center, Iowa State University, Ames, Iowa, USA

^3^Interdepartmental Plant Biology Major, Iowa State, University, Ames, Iowa, USA

^4^Interdepartmental Genetics and Genomics Major, Iowa State, University, Ames, Iowa, USA

^5^Department of Biological Sciences, University of Missouri, Columbia, Missouri, USA

*****Corresponding Author,

kanwang@iastate.edu

### Media:

**For doubled haploid treatment:**

MS Rooting Medium plus colchicine:

|  | ****Chemicals**** | ****Final concentration**** |
| --- | --- | --- |
| **1.** | **MS Basal Medium Powder** | **4.43 g/L** |
| **2.** | **Sucrose** | **30 g/L** |
|  | ***Adjust pH to 5.8, then add*** |  |
| **3.** | **Gelrite** | **2.5 g/L** |
|  | ***Autoclave, cool down to 55 ^o^C, then add*** |  |
| **4.** | **Carbinicillin (filter sterilized)** | **100 mg/L** |
| **5.** | **Colchicine (filter sterilized)** | **0.05% (w/v)** |

Callus Development Medium (605J) (Lowe et al., 2016):

|  | ****Chemicals**** | ****Final concentration**** |
| --- | --- | --- |
| **1.** | **MS Basal Salt Mixture** | **4.3 g/L** |
| **2.** | **N6 Macro Nutrient Stock^1^** | **60 mL/L** |
| **3.** | **B5 Micro Nutrient Stock^2^** | **0.6 mL/L** |
| **4.** | Eriksson’s Vitamins | 0.4 mL/L |
| **5.** | Schenk and Hildebrandt Vitamin Powder | 0.6 g/L |
| **6.** | Ferrous Sodium Stock^3^ | 6 mL/L |
| **7.** | KNO_3_ | 1.68 g/L |
| **8.** | Thiamine HCl | 0.2 g/L |
| **9.** | Casein Hydrolysate | 0.3 g/L |
| **10.** | 2,4-Dichlorophenoxyacetic acid (2,4-D) | 0.8 mg/L |
| **11.** | L-proline | 2.0 g/L |
| **12.** | **Sucrose** | **20 g/L** |
| **13.** | Glucose | 0.6 g/L |
|  | ***Adjust pH to 5.8, then add*** |  |
| **14.** | Noble agar | **8 g/L** |
|  | ***Autoclave, cool down to 55 ^o^C, then add*** |  |
| **15.** | Dicamba (filter sterilized) | 1.2 mg/L |
| **16.** | **Carbenicillin (filter sterilized)** | **100 mg/L** |

**1.** N6 Macro Nutrient Stock: CaCl**_2_**•2H**_2_**O, 1.66 g/L; (NH₄)**_2_**SO**_4_,** 4.62 g/L; KH**_2_**PO**_4_,** 4 g/L; MgSO**_4_** •7H**_2_**O, 1.85 g/L; KNO_3_, 28.3 g/L.

**2. B5 Micro Nutrient Stock: Boric Acid, 3 g/L; MnSO_4_•H_2_O, 10 g/L; Na_2_MoO_4_•2H_2_O, 0.25 g/L; KI, 0.75 g/L.**

**3. EDTA-Na_2_•2H_2_O, 3.7 g/L; FeSO4•7H_2_O, 2.79 g/L.**

Shoot Formation Medium (289O) (Lowe et al., 2016):

|  | ****Chemicals**** | ****Final concentration**** |
| --- | --- | --- |
| **1.** | **MS Basal Medium Powder** | **4.43 g/L** |
| **2.** | Zeatin | 0.5 mg/L |
| **3.** | **Sucrose** | **60 g/L** |
|  | ***Adjust pH to 5.8, then add*** |  |
| **4.** | Noble agar | **8 g/L** |
|  | ***Autoclave, cool down to 55 ^o^C, then add*** |  |
| **5.** | Thidiazuron (filter sterilized) | 0.1 mg/L |
| **6.** | 6-Benzylaminopurine (BAP, filter sterilized) | 1 mg/L |
| **7.** | **Carbenicillin (filter sterilized)** | **100 mg/L** |

**For FFMM-AT transformation:**

YP Medium plus thymidine:

|  | ****Chemicals**** | ****Final concentration**** |
| --- | --- | --- |
| **1.** | **Pepton** | **10 g/L** |
| **2.** | Yeast Extract | 5 g/L |
| **3.** | **NaCl** | **5 g/L** |
|  | ***Adjust pH to 6.8, then add*** |  |
| **4.** | Bacto agar | **15 g/L** |
|  | ***Autoclave, cool down to 55 C, then add*** |  |
| **5.** | Thymidine (filter sterilized) | 50 mg/L |
| **6.** | Kanamycin (filter sterilized) | 100 mg/L |
| **7.** | **Gentamicin (filter sterilized)** | **50 mg/L** |

**Liquid infection medium (700A) (Jones et al., 2019; Masters et al., 2020):**

|  | ****Chemicals**** | ****Final concentration**** |
| --- | --- | --- |
| **1.** | **MS Basal Medium Powder** | **4.43 g/L** |
| **2.** | 2,4-Dichlorophenoxyacetic acid (2,4-D) | 1.5 mg/L |
| **3.** | **Sucrose** | **68.5 g/L** |
| **4.** | **Glucose** | **36 g/L** |
|  | ***Adjust pH to 5.8, filter sterilize, then add*** |  |
| **5.** | Thymidine (filter sterilized) | 50 mg/L |

**Cocultivation medium (562V) (Jones et al., 2019; Masters et al., 2020):**

|  | ****Chemicals**** | ****Final concentration**** |
| --- | --- | --- |
| **1.** | **N6 Basal Salt Mixture** | **4.3 g/L** |
| **2.** | 2,4-Dichlorophenoxyacetic acid (2,4-D) | 2.0 mg/L |
| **3.** | **Sucrose** | **30 g/L** |
|  | ***Adjust pH to 5.8, then add*** |  |
| **4.** | Noble agar | **8 g/L** |
|  | ***Autoclave, cool down to 55 ^o^C, then add*** |  |
| **5.** | Thymidine (filter sterilized) | 50 mg/L |
| **6.** | Silver Nitrate **(filter sterilized)** | **1 mg/L** |
| **7.** | **Acetosyringone** | **100** µM |

**Resting Medium (605T) (Lowe et al., 2016):**

|  | ****Chemicals**** | ****Final concentration**** |
| --- | --- | --- |
| **1.** | **MS Basal Salt Mixture** | **4.3 g/L** |
| **2.** | **N6 Macro Nutrient Stock^1^** | **60 mL/L** |
| **3.** | **B5 Micro Nutrient Stock^2^** | **0.6 mL/L** |
| **4.** | Eriksson’s Vitamins | 0.4 mL/L |
| **5.** | Schenk and Hildebrandt Vitamin Powder | 0.6 g/L |
| **6.** | Ferrous Sodium Stock^3^ | 6 mL/L |
| **7.** | KNO_3_ | 1.68 g/L |
| **8.** | Thiamine HCl | 0.2 g/L |
| **9.** | Casein Hydrolysate | 0.3 g/L |
| **10.** | 2,4-Dichlorophenoxyacetic acid (2,4-D) | 0.8 mg/L |
| **11.** | L-proline | 2.0 g/L |
| **12.** | **Sucrose** | **20 g/L** |
| **13.** | Glucose | 0.6 g/L |
|  | ***Adjust pH to 5.8, then add*** |  |
| **14.** | Noble agar | **8 g/L** |
|  | ***Autoclave, cool down to 55 ^o^C, then add*** |  |
| **15.** | Dicamba (filter sterilized) | 1.2 mg/L |
| **16.** | **Cefotaxime (filter sterilized)** | **100 mg/L** |
| **17.** | **Timentin (filter sterilized)** | **150 mg/L** |

**1.** N6 Macro Nutrient Stock: CaCl**_2_**•2H**_2_**O, 1.66 g/L; (NH₄)**_2_**SO**_4_,** 4.62 g/L; KH**_2_**PO**_4_,** 4 g/L; MgSO**_4_** •7H**_2_**O, 1.85 g/L; KNO_3_, 28.3 g/L.

**2. B5 Micro Nutrient Stock: Boric Acid, 3 g/L; MnSO_4_•H_2_O, 10 g/L; Na_2_MoO_4_•2H_2_O, 0.25 g/L; KI, 0.75 g/L.**

**3. EDTA-Na_2_•2H_2_O, 3.7 g/L; FeSO4•7H_2_O, 2.79 g/L.**

Selection Medium I or II (modified 605J plus bialaphos):

|  | ****Chemicals**** | ****Final concentration**** |
| --- | --- | --- |
| **1.** | **MS Basal Salt Mixture** | **4.3 g/L** |
| **2.** | **N6 Macro Nutrient Stock^1^** | **60 mL/L** |
| **3.** | **B5 Micro Nutrient Stock^2^** | **0.6 mL/L** |
| **4.** | Eriksson’s Vitamins | 0.4 mL/L |
| **5.** | Schenk and Hildebrandt Vitamin Powder | 0.6 g/L |
| **6.** | Ferrous Sodium Stock^3^ | 6 mL/L |
| **7.** | KNO_3_ | 1.68 g/L |
| **8.** | Thiamine HCl | 0.2 g/L |
| **9.** | Casein Hydrolysate | 0.3 g/L |
| **10.** | 2,4-Dichlorophenoxyacetic acid (2,4-D) | 0.8 mg/L |
| **11.** | L-proline | 2.0 g/L |
| **12.** | **Sucrose** | **20 g/L** |
| **13.** | Glucose | 0.6 g/L |
| **14.** | 2-(*N*-morpholino)ethanesulfonic acid (MES) | 0.5 g/L |
|  | ***Adjust pH to 5.8, then add*** |  |
| **15.** | Noble agar | **8 g/L** |
|  | ***Autoclave, cool down to 55 ^o^C, then add*** |  |
| **16.** | Dicamba (filter sterilized) | 1.2 mg/L |
| **17.** | **Carbenicillin (filter sterilized)** | **100 mg/L** |
| **18a.** | **Bialaphos (filter sterilized), for Selection I** | **3 mg/L** |
| **18b.** | **Bialaphos (filter sterilized), for Selection II** | **6 mg/L** |

**1.** N6 Macro Nutrient Stock: CaCl**_2_**•2H**_2_**O, 1.66 g/L; (NH₄)**_2_**SO**_4_,** 4.62 g/L; KH**_2_**PO**_4_,** 4 g/L; MgSO**_4_** •7H**_2_**O, 1.85 g/L; KNO_3_, 28.3 g/L.

**2. B5 Micro Nutrient Stock: Boric Acid, 3 g/L; MnSO_4_•H_2_O, 10 g/L; Na_2_MoO_4_•2H_2_O, 0.25 g/L; KI, 0.75 g/L.**

**3. EDTA-Na_2_•2H_2_O, 3.7 g/L; FeSO4•7H_2_O, 2.79 g/L.**

Shoot Formation Medium (289O) plus bialaphos (Lowe et al., 2016):

|  | ****Chemicals**** | ****Final concentration**** |
| --- | --- | --- |
| **1.** | **MS Basal Medium Powder** | **4.43 g/L** |
| **2.** | Zeatin | 0.5 mg/L |
| **3.** | **Sucrose** | **60 g/L** |
|  | ***Adjust pH to 5.8, then add*** |  |
| **4.** | Noble agar | **8 g/L** |
|  | ***Autoclave, cool down to 55 ^o^C, then add*** |  |
| **5.** | Thidiazuron (filter sterilized) | 0.1 mg/L |
| **6.** | 6-Benzylaminopurine (BAP, filter sterilized) | 1 mg/L |
| **7.** | **Carbenicillin (filter sterilized)** | **100 mg/L** |
| **8.** | **Bialaphos (filter sterilized)** | **3 mg/L** |

Rooting Medium plus bialaphos (modified from Lowe et al., 2016):

|  | ****Chemicals**** | ****Final concentration**** |
| --- | --- | --- |
| **1.** | **MS Basal Medium Powder** | **4.43 g/L** |
| **2.** | **Sucrose** | **40 g/L** |
| **3.** | 2-(*N*-morpholino)ethanesulfonic acid (MES) | 0.5 g/L |
|  | ***Adjust pH to 5.8, then add*** |  |
| **4.** | Gelrite | **3 g/L** |
|  | ***Autoclave, cool down to 55 ^o^C, then add*** |  |
| **5.** | **Bialaphos (filter sterilized)** | **2 mg/L** |

**References:**

Jones T., Lowe, K., Hoerster, G., Anand, A., Wu, E., Wang, N., et al. (2019). “Maize transformation using the morphogenic genes Baby Boom and Wuschel2,” in *Transgenic Plants*. Eds. S. Kumar, P. Barone, M. Smith (New York, NY, Humana Press), pp 81-93.

Lowe, K., Wu, E., Wang, N., Hoerster, G., Hastings, C., Cho, M.-J., et al. (2016). Morphogenic regulators Baby Boom and Wuschel improve monocot transformation, *Plant Cell* 28, 1998-2015.

Masters, A., Kang, M., McCaw, M., Zobrist, J., Gordon-Kamm, W., Jones, T., et al. (2020). *Agrobacterium*-mediated immature embryo transformation of recalcitrant maize inbred lines using morphogenic genes. *J. Vis. Exp*. (156), e60782. doi: 10.3791/60782.


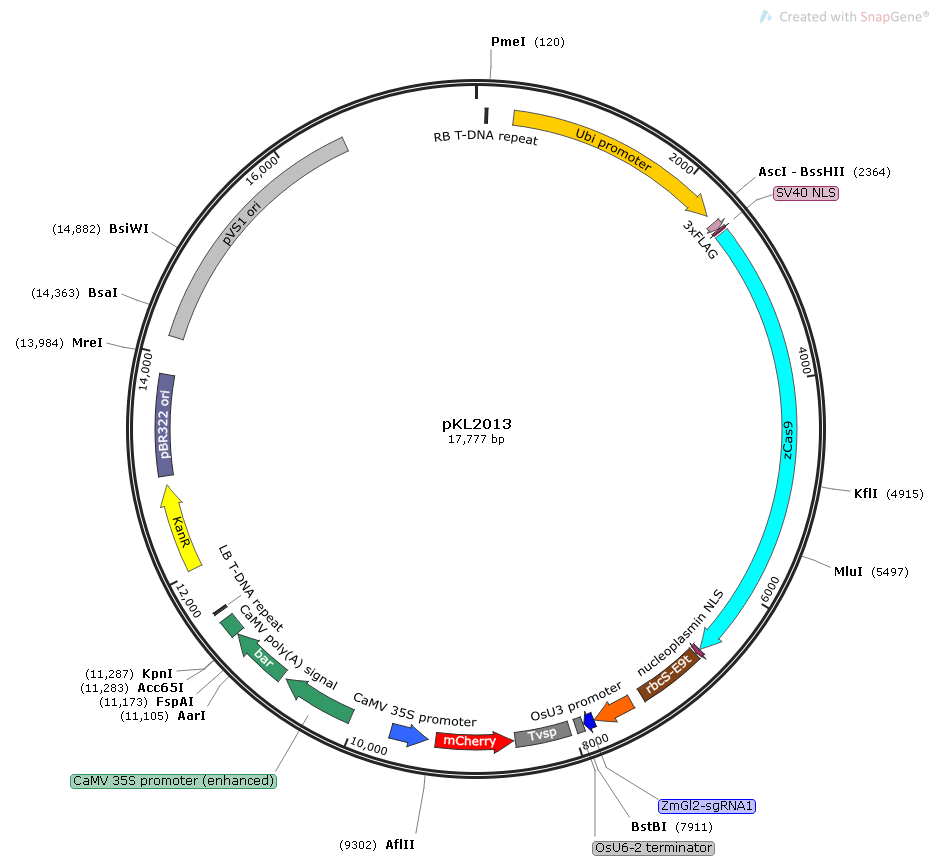


**Figure S1.** Map of pKL2013.
